# Chloroquine Inhibits Dengue Virus Type 2 Replication in Vero Cells but Not in C6/36 Cells

**DOI:** 10.1155/2013/282734

**Published:** 2013-01-31

**Authors:** Kleber Juvenal Silva Farias, Paula Renata Lima Machado, Benedito Antônio Lopes da Fonseca

**Affiliations:** ^1^Department of Internal Medicine, School of Medicine of Ribeirão Preto, University of São Paulo, 14049-900 Ribeirão Preto, SP, Brazil; ^2^Program of Graduate Studies on Applied Microbiology and Immunology, School of Medicine of Ribeirão Preto, University of São Paulo, 14049-900 Ribeirão Preto, SP, Brazil

## Abstract

Dengue viruses are the most important arthropod-borne viruses in terms of morbidity and mortality in the world. Since there is no dengue vaccine available for human use, we have set out to investigate the use of chloroquine as an antiviral drug against dengue. Chloroquine, an amine acidotropic drug known to affect intracellular exocytic pathways by increasing endosomal pH, was used in the in vitro treatment of Vero and C6/36 cells infected with dengue virus type 2 (DENV-2). Real-time RT-PCR and plaque assays were used to quantify the DENV-2 load in infected Vero and C6/36 cells after chloroquine treatment. Our results showed that a dose of 50 **μ**g/ml of chloroquine was not toxic to the cells and induced a statistically significant inhibition of virus production in infected Vero cells when compared to untreated cells. In C6/36 cells, chloroquine does not induce a statistically significant difference in viral replication when compared to untreated cells, showing that this virus uses an unlikely pathway of penetration in these cells, and results were also confirmed by the plaque assay (PFU). These data suggest that the inhibition of virus infection induced by chloroquine is due to interference with acidic vesicles in mammalian cells.

## 1. Introduction

 Dengue viruses (DENV) are single-stranded, positive-sense RNA viruses that belong to the genus *Flavivirus *of the family *Flaviviridae*. They consist of four serotypes (from DENV-1 to DENV-4) that are transmitted to humans and other higher primates by mosquitoes of the genus *Aedes *[1]. DENV cause the most important arthropod-borne viral disease in the world, characterized by a wide spectrum of clinical manifestations ranging from a flu-like disease (dengue fever) to a life-threatening disease known as dengue hemorrhagic fever/dengue shock syndrome (DHF/DSS). The only available way to control dengue is vector control since there is neither a vaccine for human use nor an antiviral drug to inhibit virus replication. Little is known about the site of flavivirus assembly or the details of maturation, but a decrease in endosomal pH is necessary for flavivirus envelope fusion with endosomal membranes leading to release of the viral capsid into the cytosol and initiation of virus replication [2]. Weak bases such as chloroquine (CLQ) accumulate in acidic vesicles and raise the pH therein. By preventing the low pH-induced fusion of viral envelope and cell endosome membranes, CLQ is potentially able to block the entry of certain viruses into the cytosol [3]. The present study examined the effect of CLQ on the DENV-2 replication in Vero (mammalian) and C6/36 (mosquito) cells in order to define whether or not CLQ could be used to reduce viral yield.

## 2. Materials and Methods

### 2.1. Cell Cultures

 Vero cells (continuous cell lineage originated from the kidney of African green monkeys) were grown in Leibovitz-15 (L-15) culture medium (Invitrogen, New York, USA) supplemented with 10% Fetal Bovine Serum (FBS), 1% L-glutamine 200 mM, 1% penicillin G (100 U/mL), streptomycin (100 *μ*g/mL) and 10% triptose phosphate. Vero cells were maintained at 37°C and 5% CO_2_. C6/36, an *Aedes albopictus *cell line, was cultured in L-15 medium supplemented with 10% FBS, L-glutamine, 10% triptose phosphate, 1% penicillin G (100 U/mL), streptomycin (100 *μ*g/mL), at 28°C in the absence of CO_2_.

### 2.2. Virus Stock

 DENV-2 New Guinea C strain, recovered from the brain of newborn Swiss mice, was used in this study. The viral stock was prepared by inoculation of C6/36 cells contained in 75 cm^2^ culture flasks with virus diluted in 1 mL of L-15-2% FBS. After 1 h, 14 mL of L-15 supplemented with 10% FBS was added, and the cells were cultured for 7 days. Cells culture supernatant was then harvested and centrifuged at 2,000 Xg for 5 min to removed cell debris. The supernatant containing the virus was adjusted to 20% FBS, aliquoted, and stored at −70°C. Virus stock and cell culture supernatants used in the present study were free of the lipopolysaccharide and mycoplasma.

DENV-2 was used in the study because it showed the best of titration in preliminary experiments to assess the antiviral effect of chloroquine in Vero and C6/36 cells.

### 2.3. Dengue Virus Titration

Virus production was titrated by plaque assay using Vero cells. Vero cells were seeded in 12-well (6 × 10^5^ cells/well) plate in L-15 medium with 10% FBS for 48 h at 37°C. Medium was removed, and decimal serial dilutions of virus stock or supernatant of cells treated with CLQ, prepared in L-15 medium with 2% FBS, were added (0.1 mL/well) to the cells, which were then incubated for 2 h at 37°C. Subsequently, L-15 medium containing 5% FBS and 3% carboxymethyl-cellulose (1 mL/well) (overlay) was added, and the plate was incubated at 37°C for 7 days. Overlay was removed on day seven, and cells were fixed with a solution of 10% formaldehyde in PBS. After 2 hours at room temperature, the formaldehyde solution was removed, and cells were washed twice with PBS and stained (15 min) with a 1% crystal violet solution in 20% ethanol. The plaques of cell lysis were counted, and the virus concentration was expressed as plaque forming unites (PFU) per milliliter.

### 2.4. Chloroquine Cytotoxic Assay

 Cytotoxicity induced by CLQ was assayed in Vero and C6/36 cells to determine the ideal concentration for the experiments. Vero and C6/36 cells were seeded in 24-well (4 × 10^5^ cells/well) plates. After an incubation period of approximately 72 hours, the culture medium was replaced with L-15 medium containing 2% FBS and different concentrations of CLQ (Sigma-Aldrich, USA). After incubation periods of 1, 6, 12, 24, 48, 72, and 96 hours with the drugs, the cells were removed with trypsin, and viability of Vero and C6/36 cells was confirmed by the Trypan blue exclusion method (Invitrogen, New York, USA).

### 2.5. Treatment of DENV-2 Infected Cells with CLQ

 Monolayers of Vero and C6/36 cells seeded onto 24-well plates were infected with DENV-2 at a multiplicity of infection (MOI) of 0.1. At 1 h postinfection (p.i.) the inoculum was removed. Vero and C6/36 cells were incubated in the presence of different concentrations of CLQ (0; 0.05; 0.5; 5; 50 *μ*g/mL) at defined time intervals, in different experiments: (i) concomitantly, 1 h after infection; (ii) concomitantly, 1 h after infection, and at 24-hour intervals for 7 days; (iii) concomitantly, 1 hour after infection and at 12-hour intervals for 7 days. DENV-2 replication of mock control was 0 *μ*g/mL. Infected cell supernatants were collected at 0, 6, 12, 24, 48, 72, 96, 120, 144, and 168 hours postinfection, cleared by centrifugation, and stored in aliquots at −70°C until use in real-time PCR and plaque assays. The experiments were carried out in duplicate, and the results are shown as the mean values obtained from three individual experiments.

### 2.6. Viral RNA Extraction

 Viral RNA was extracted from 140 *μ*L of each supernatant sample using the QIAamp Viral RNA kit (QIAGEN, USA) according to manufacturer's directions.

### 2.7. Real-Time Quantitative Reverse Transcriptase-Polymerase Chain Reaction (qRT-PCR) Assay

 The real-time qRT-PCR to determine the number of DENV-2 RNA copies present in each supernatant was carried out as described previously [4]. Briefly, to construct the standard curve, the number of DENV-2 particles produced by infected cells was measured as the number of RNA copies quantitated by qRT-PCR in serially diluted infected cell supernatants. Each qRT-PCR contained 12.5 *μ*L of the Sybr Green Master Mix reagent (Applied Biosystems), 0.5 *μ*L of RNase inhibitor, 0.13 *μ*L of multiscribe (50 U/*μ*L), 0.5 *μ*L of primers (20 nM) DV2U (5′-AAGGTGAGATGAAGCTGTAGTCTC-3′), and DVL1 (5′-CATTCCATTTTCTGGCGTTCT-3′) [5] specifically designed to anneal to the DENV-2 3′untranslated region (3′-UTR), 5.87 *μ*L of DEPC water, and 5 *μ*L of RNA to a final volume of 25 *μ*L. The amplification protocol consisted of the following steps: 48°C for 30 min, 95°C for 10 min, followed by 40 cycles at 95°C for 15 seconds, and finally 60°C for 1 min. The same protocol was used to quantify the DENV-2 RNA copies present in the supernatants of CLQ-treated and untreated Vero and C6/36 cells infected with DENV-2, collected at defined postinfection intervals, as described above.

### 2.8. Statistical Analysis

 Statistical analysis was used to assess the difference in viral yield, at time-defined intervals, produced in infected cells in contact with CLQ compared to control cells (without the drug). Data were entered into the GraphPad Prism software, version 3.0 (GraphPad Software Inc., EUA), and submitted to one-way ANOVA (nonparametric test) analysis followed by the Bonferroni test. For all analyses, values of *P* < 0.05 were considered statistically significant.

## 3. Results

### 3.1. Cytotoxicity of CLQ in C6/36 and Vero Cells

**Figure 1:**
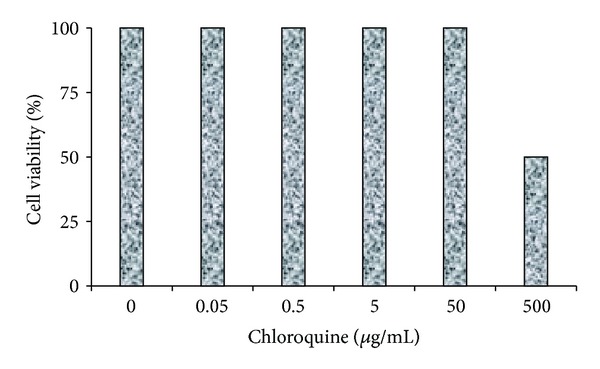
The effect of CLQ on the cytotoxicity in C6/36 cells. Concentrations equal to or higher than 500 *μ*g/mL (CLQ) were highly cytotoxic to C6/36 cells, while concentrations equal to or lower than 50 *μ*g/mL (CLQ) did not induce significant cytotoxicity.

**Figure 2:**
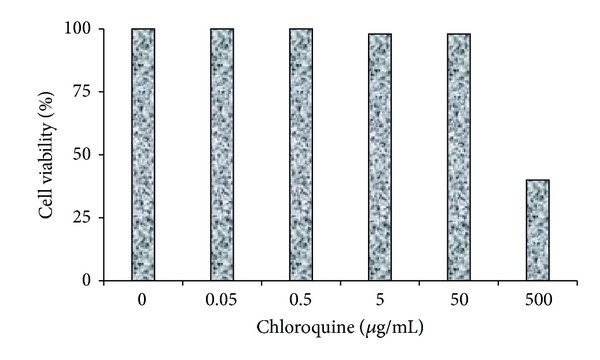
The effect of CLQ on the cytotoxicity in Vero cells. Concentrations equal to or higher than 500 *μ*g/mL (CLQ) were highly cytotoxic to Vero cells, while concentrations equal to or lower than 50 *μ*g/mL (CLQ) did not induce significant cytotoxicity.

 CLQ was highly cytotoxic to C6/36 and Vero cells when they were treated with a concentration equal to or higher than 500 *μ*g/mL, while no significant cytotoxicity was observed when the cells were treated with a concentration equal to or lower than 50 *μ*g/mL (Figures 1 and 2). Based on these data, CLQ was used in concentrations equal to or lower of 50 *μ*g/mL used in the experiments.

### 3.2. Effect of CLQ on DENV-2 Replication

**Figure 3:**
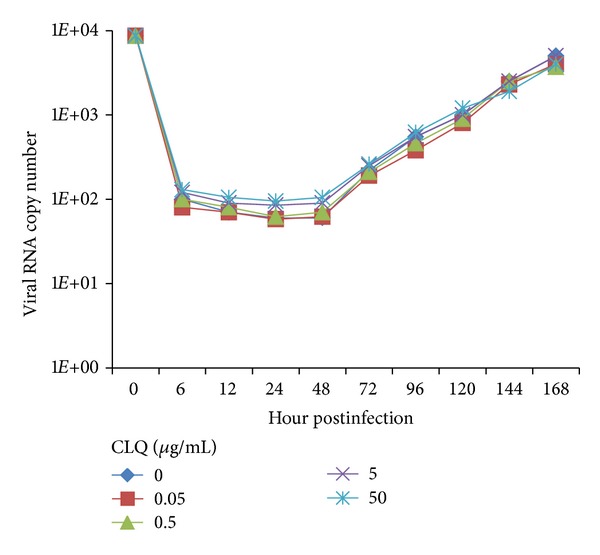
Action of chloroquine on DENV-2 replication in C6/36 cells. The viral RNA present in the culture supernatants of C6/36 cells infected with DENV-2, both untreated and treated with chloroquine just after infection, was extracted and analyzed by qRT-PCR. The results represent the average values of the number of copies of viral RNA (*P* > 0.05).

**Figure 4:**
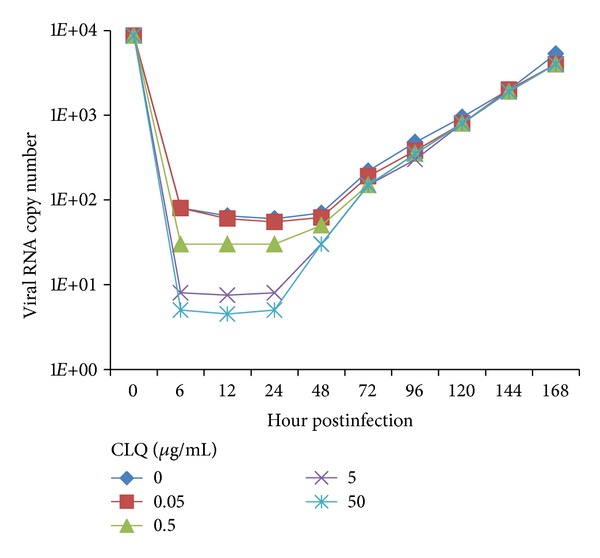
Action of chloroquine on DENV-2 replication in Vero cells. The viral RNA present in the culture supernatants of Vero cells infected with DENV-2, both untreated and treated with chloroquine just after infection, was extracted and analyzed by qRT-PCR. The results represent the average values of the number of copies of viral RNA (*P* < 0.001).

 To determine whether CLQ would inhibit DENV-2 replication, monolayers of Vero and C6/36 cells were infected with DENV-2 and incubated with 2% FBS L-15 medium containing different concentrations of the drug; then the virus production was quantified by qRT-PCR and plaque assay. CLQ had no effect on DENV-2 replication in C6/36 cells (Figure 3), but showed a dose-dependent inhibition of the virus replication in Vero cells when analyzed by qRT-PCR (Figure 4). The viral replication in Vero cells was significantly reduced by the addition of ≥5 *μ*g/mL of CLQ 1 h after infection when compared to untreated cells; this inhibition was maintained up to 24 h after infection (Figure 4). The same results were obtained when the analysis was carried out by plaque assay with an excellent correlation with the qRT-PCR (data not shown).

The viral inhibition effect induced by CLQ for only 24 h may be related to the consumption of the drug. To further analyze the antiviral effect of CLQ, Vero cells were treated with the drug 1 hour after infection and at 12- and 24-hour intervals after infection.

**Figure 5:**
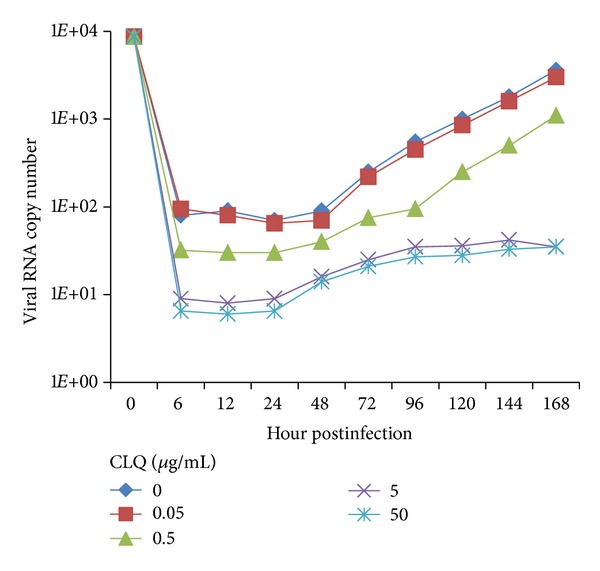
Action of chloroquine added at 24-hour intervals on DENV-2 replication in Vero cells. The viral RNA present in the culture supernatants of Vero cells infected with DENV-2, both untreated and treated chloroquine (every 24 hours after infection), was extracted and analyzed by qRT-PCR. The results represent the average values of the number of copies of viral RNA (*P* < 0.05).

**Figure 6:**
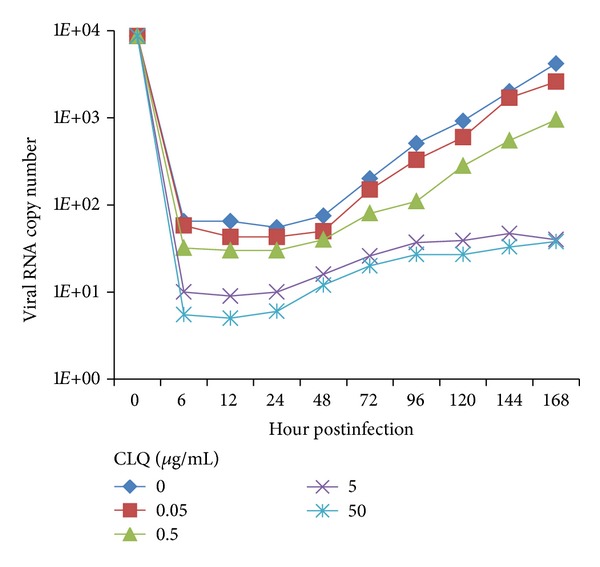
Action of chloroquine added at 12-hour intervals on DENV-2 replication in Vero cells. The viral RNA present in the culture supernatants of Vero cells infected with DENV-2, both untreated and treated chloroquine (every 12 hours after infection), was extracted and analyzed by qRT-PCR. The results represent the average values of the number of copies of viral RNA (*P* < 0.01).

 The qRT-PCR showed a significant reduction in viral yield compared to untreated cells up to the 7th day after infection (Figures 5 and 6). The analysis by plaque assay was again in agreement with qRT-PCR (data not shown).

 Viral replication in Vero-infected cells was impaired by the addition of CLQ at 1 hour after infection and at 12-hour intervals after infection. With this approach, both the plaque assay and qRT-PCR showed a statistically significant reduction in viral yield similar to obtained for cells exposed to CLQ at 24-hour intervals.

## 4. Discussion

 We report here data showing that CLQ is an effective antiviral agent for DENV-2 under cell culture condition. The inhibitory effect was observed when the drug was added 1 h after the initiation of infection, probably due to the increase of the endosomes pH and thus subverting the ongoing fusion events between virus envelope and endosome membranes. These results suggest that CLQ could have a potential for therapeutic use against dengue virus and even against other viruses that penetrate cells by endocytosis. In that sense, some studies have shown that CLQ inhibits SARS coronavirus (SARS-CoV) replication in vitro [6, 7]. In addition, di Trani et al. [8] tested the antiviral effects of CLQ in vitro against selected human and avian viruses belonging to different subtypes and displaying different pH requirements. Those authors found that CLQ had inhibitory effect against the viruses when the drug had been added at the time of infection and the effect was lost after 2-hour postinfection. Finally, Eng et al. [9] reported that CLQ is able to inhibit influenza A virus replication in vitro.

 CLQ is a safe drug with over half a century of use in the treatment of malaria; therefore, the implementation of a protocol to use it in dengue treatment would not be a problem. Since there is a correlation between high viral load and development of DHF/DSS [10], the use of CLQ to treat dengue virus infection as soon as the patients present the dengue symptoms could prevent the development of the severe forms of the disease due to the reduction of dengue viremia. Furthermore, due to its interference with TNF-*α* cytotoxicity [11], CLQ would probably reduce the inflammatory symptoms, such as high fever and backache, associated with dengue.

 The inhibitory effect of CLQ on virus replication in mammalian cells has already been studied with other viruses. This drug has been used to control infection with coronavirus (SARS-CV), the etiologic agent of severe acute respiratory syndrome (SARS) in nonhuman primate cells (Vero) [7]. It was also used to inhibit the replication of human immunodeficiency virus (HIV-1) in lineages of monocytes and lymphocytes [12] and lineages of H9 cells [13]. Furthermore, CLQ has been used to inhibit Kunjin virus replication in cells of nonhuman primates (Vero) [14].

 Dengue viruses replicate well in C6/36 cells and in other mosquito lines, they are presently used as sensitive assays for virus isolation from patients, and Vero are also permissive cell lines [15]. In the present study, we observed that in experiments carried out with C6/36 cells infected with DENV-2 and treated with CLQ did not reduce the viral load. In 1981, Coombs et al. [16] observed also that CLQ did not reduce the viral load in experiments carried out with the Sindbis virus in cells of the mosquito *Aedes albopictus*. Hernandez et al. [17] obtained similar results when they used CLQ to inhibit replication of the Sindbis virus. Two explanations are possible for these findings: (i) CLQ does not block endosome acidification in the cells of the mosquito *Aedes albopictus*, or (ii) CLQ blocks endosome acidification but not the infection of mosquito cells with the Sindbis virus. These facts suggest that the replication of DENV-2 used in our experiments in *Aedes albopictus* cells occurs possibly by a pathway of intracytoplasmic penetration other than the endosomal one, which was not blocked by CLQ.

 Taken together with the data obtained in these studies, our results suggest that CLQ interferes in DENV-2 virus replication in Vero cells culture but not in C6/36 cells.

 Thus, as chloroquine is considered a safe and effective drug, used to treatment many diseases, including malaria, their therapeutic use is promising because it was shown in this study that the drug has significant antiviral effect on the replication of dengue-2 virus in cells culture.

## References

[1] da Fonseca BAL, Fonseca SNS (2002). Dengue virus infections. *Current Opinion in Pediatrics*.

[2] Lindenbach BD, Rice CM (2003). Molecular biology of flaviviruses. *Advances in Virus Research*.

[3] de Duve C (1983). Lysosomes revisited. *European Journal of Biochemistry*.

[4] Gomes-Ruiz AC, Nascimento RT, de Paula SO, da Fonseca BAL (2006). SYBR green and TaqMan real-time PCR assays are equivalent for the diagnosis of dengue virus type 3 infections. *Journal of Medical Virology*.

[5] Houng HSH, Chen RC, Vaughn DW, Kanesa-thasan N (2001). Development of a fluorogenic RT-PCR system for quantitative identification of dengue virus serotypes 1–4 using conserved and serotype-specific 3' noncoding sequences. *Journal of Virological Methods*.

[6] Keyaerts E, Vijgen L, Maes P, Neyts J, Ranst MV (2004). In vitro inhibition of severe acute respiratory syndrome coronavirus by chloroquine. *Biochemical and Biophysical Research Communications*.

[7] Vincent MJ, Bergeron E, Benjannet S (2005). Chloroquine is a potent inhibitor of SARS coronavirus infection and spread. *Virology Journal*.

[8] di Trani L, Savarino A, Campitelli L (2007). Different pH requirements are associated with divergent inhibitory effects of chloroquine on human and avian influenza A viruses. *Virology Journal*.

[9] Eng EO, Chew JSW, Jin PL, Chua RCS (2006). In vitro inhibition of human influenza A virus replication by chloroquine. *Virology Journal*.

[10] Wang WK, Chao DY, Kao CL (2003). High levels of plasma dengue viral load during defervescence in patients with dengue hemorrhagic fever: implications for pathogenesis. *Virology*.

[11] Kull FC (1988). The TNF receptor in TNF-mediated cytotoxicity. *Natural Immunity and Cell Growth Regulation*.

[12] Sperber K, Kalb TH, Stecher VJ, Banerjee R, Mayer L (1993). Inhibition of human immunodeficiency virus type 1 replication by hydroxychloroquine in T cells and monocytes. *AIDS Research and Human Retroviruses*.

[13] Tsai WP, Nara PL, Kung HF, Oroszlan S (1990). Inhibition of human immunodeficiency virus infectivity by chloroquine. *AIDS Research and Human Retroviruses*.

[14] Mackenzie JM, Westaway EG (2001). Assembly and maturation of the flavivirus Kunjin virus appear to occur in the rough endoplasmic reticulum and along the secretory pathway, respectively. *Journal of Virology*.

[15] Gubler DJ (1998). Dengue and dengue hemorrhagic fever. *Clinical Microbiology Reviews*.

[16] Coombs K, Mann E, Edwards J, Brown DT (1981). Effects of chloroquine and cytochalasin B on the infection of cells by Sindbis virus and vesicular stomatitis virus. *Journal of Virology*.

[17] Hernandez R, Luo T, Brown DT (2001). Exposure to low pH is not required for penetration of mosquito cells by Sindbis virus. *Journal of Virology*.

